# c-Jun N-terminal kinase (JNK) cooperates with Gsk3β to regulate Dishevelled-mediated microtubule stability

**DOI:** 10.1186/1471-2121-8-27

**Published:** 2007-07-03

**Authors:** Lorenza Ciani, Patricia C Salinas

**Affiliations:** 1 Department of Anatomy and Developmental Biology, University College London, WC1E 6BT, London, UK

## Abstract

**Background:**

Wnt factors are a large family of signaling molecules that play important roles in the regulation of cell fate specification, tissue polarity and cell movement. In the nervous system, Wnts also regulates the formation of neuronal connection acting as retrograde signals that regulate the remodeling of the axons prior to the assembly of the presynaptic apparatus. The scaffold protein Dishevelled (Dvl) mimics the effect of Wnt on the neuronal cytoskeleton by increasing the number of stable microtubule along the axon shaft and inducing the formation of looped microtubules (MT) at enlarged growth cones. A divergent Wnt-Dvl canonical pathway which bifurcates downstream of Gsk3β regulates MT dynamics.

**Results:**

Here we show that the Wnt pathway also activates c-Jun N-terminal kinase (JNK) to regulate MT stabilization. Although in the Wnt planar cell polarity (PCP) pathway, JNK lays downstream of Rho GTPases, these GTPases are not required for Wnt-mediated MTs stability. Epistatic analyses and pharmacological studies suggest that the Wnt-Dvl signalling regulates the dynamic of the cytoskeleton through two different pathways that lead to inhibition of Gsk3β and activation of JNK in the same cell.

**Conclusion:**

We demonstrate a novel role for JNK in Wnt-mediated MT stability. Wnt-Dvl pathway increases MT stability through a transcription independent mechanism that requires the concomitant inhibition of Gsk3β and activation of JNK. These studies demonstrate that Wnts can simultaneously activate different signalling pathways to modulate cytoskeleton dynamics.

## Background

Regulation of the MT cytoskeleton is crucial for diverse cellular functions such as cell division, cell polarity, migration and morphogenesis. In the nervous system, regulation of the dynamic and organization of MTs is essential for the initiation, extension and maintenance of neuronal processes. MT arrays are also used for the transport of organelles and molecules along axons and dendrites. MTs are dynamic polymers that can polymerize and depolymerise very rapidly and this dynamic instability allows cells to respond quickly to extracellular signals. Although great progress has been made in understanding how intracellular molecules regulate the dynamics of MTs [1-3], very little is known about the mechanisms by which extracellular signals and their pathways modulate MT behaviour.

The Wnt signalling pathway has been shown to directly regulate the cytoskeletal network by regulating both the dynamics and organization of MTs [4,5]. Wnts can function as molecules that guide axons to their appropriate targets [6-8] but they can also function as target-derived signals to regulate the terminal arborisation of axons [9,10]. In the latter case, Wnt proteins act retrogradely to inhibit axon extension and to induce the terminal remodelling of incoming presynaptic axons. As target-derived signals, Wnts elicit profound changes in the organization and stability of MTs of remodelled axons. Wnts induce unbundling of MTs and the formation of looped MTs at the distal portion of the axon [9,11]. Concomitantly, Wnts increase the stability of MTs manifested by an increased number of stable population (acetylated) of MTs and an increased resistance to MT depolymerising drugs [4,5]. Although initial characterization demonstrated a role for Gsk3β, a serine/threonine kinase [4], the mechanism by which Wnt signalling regulates the organization and stability of MTs remains poorly understood.

The signalling pathways activated by Wnts are well characterized. Binding of Wnt proteins to their Frizzled receptors activates the cytoplasmic scaffold protein Dvl. Downstream of Dvl, three main Wnt pathways can be activated. In the canonical Wnt signalling pathway, Dvl induces the disruption of the cytoplasmic complex formed by APC, AXIN, Gsk3β and β-catenin. Dvl inhibits Gsk3β resulting in increased stability of β-catenin, a direct target of Gsk3β. Accumulation of β-catenin leads to its translocation to the nucleus where, upon binding to the transcription factors LEF or TCF, it stimulates the transcription of target genes [12]. Wnts can also signal through the PCP pathway in which Dvl activates small Rho GTPases resulting in the activation of JNK [13]. In addition, Wnt proteins can signal through a calcium pathway that requires calcium mobilization and activation of PKC [13]. Thus, Dvl modulates the activation of the three known Wnt signalling pathways. What determines the activation of a specific pathway is not well understood but several studies suggest that different Wnts and their receptors confer this specificity. Although activation of these pathways have been observed in different cellular and developmental contexts, it remains plausible that Wnt proteins could activate two or more pathways in the same cell and that different branches of the Wnt pathway might interact with one another.

A divergent canonical Wnt pathway regulates MT dynamics. We have shown that Wnt signalling increases MT stability through a pathway that requires Dvl and inhibition of Gsk3β but it bifurcates downstream of Gsk3β in a transcription independent manner [4,5]. Instead, Dvl acts locally to inhibit Gsk3β, resulting in local changes in phosphorylation levels of Gsk3β targets such as the MT-associated protein MAP1B [5]. Consistently, phosphorylation of MAP1B by Gsk3β has been implicated in MT dynamics [11,14]. Several lines of evidence support the role of Gsk3β in Dvl-mediated MT stability. Firstly, expression of Gsk3β blocks the ability of Dvl to protect MTs from depolymerization. Secondly, Dvl decreases Gsk3β mediated phosphorylation of MAP1B, a process required for proper modulation of MT stability. Finally, pharmacological and molecular inhibition of Gsk3β increases MT stability. However, Gsk3β inhibition only partially mimics the ability of Dvl to stabilize MTs. These findings led us to propose that Dvl may regulate MT stability through an additional unknown pathway [5].

To begin to characterize this additional pathway we examined the contribution of the PCP pathway. Firstly, we tested the role of small Rho GTPases such as Rho, Rac and Cdc42, which have been implicated in the regulation of MT dynamics [15-21]. Interestingly, neither Rho or Rac nor Cdc42 are required for Dvl function in MT stability. In contrast, activation of c-Jun N-terminal kinase (JNK) increases MT stability whereas its inhibition blocks the ability of Dvl to stabilize MTs. Moreover, Dvl activates endogenous JNK in developing neurons. Our results suggest that Wnt-Dvl signalling activates two parallel pathways in the same cell to regulate MT dynamics. Dvl increases MT stability by activating JNK and at the same time inhibiting Gsk3β. These results demonstrate that collaboration between two pathways contribute to Wnt-mediated function on the cytoskeleton.

## Results

### Dishevelled stabilises microtubules through inhibition of Gsk3β and a second signalling pathway

**Figure 1:**
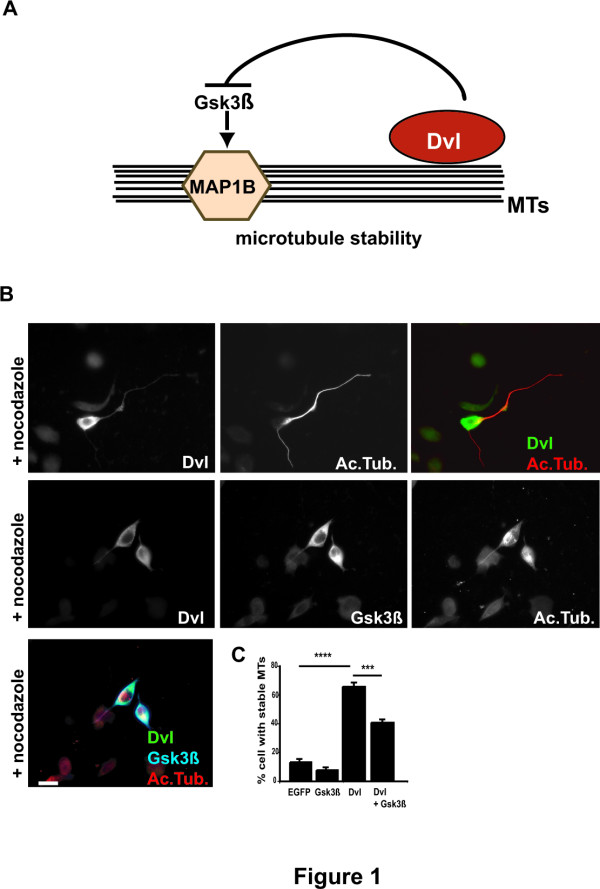
**Dishevelled stabilises microtubules through inhibition of Gsk3β and a second signalling pathway**. **A) **Diagram shows Dvl binding to the MT network and inhibiting a pool of Gsk3β resulting in changes in the phosphorylation of MAP1B and increased microtubule stability. **B) **Neurons expressing Dvl contain stable MTs after Nocodazole treatment. Expression of Gsk3β partially blocks the ability of Dvl to stabilize MTs. **C) **Quantification shows that Dvl alone induces MT stability when compared to control EGFP-expressing neurons. Neurons expressing Gsk3β alone does not induce Mt stability while neurons expressing both Gsk3β and Dvl exhibit decreased MT stability when compared to Dvl-expressing neurons. Scale bar: 15 μm. Three asterisks, *P *< 0.001; For asterisks, *P *< 0.0001.

We have previously shown that activation of the Wnt-Dvl signalling pathway increases MT stability in dividing and post-mitotic cells [4,5]. Dvl fully mimics the effect of Wnts by increasing the amount of stable MTs and inducing MT looping and axon remodelling as observed in primary neurons exposed to Wnts [5]. Our studies indicate that Dvl binds to MTs and locally inhibits a pool of the Gsk3β kinase resulting in changes in the phosphorylation of the MT-associated protein MAP1B (Fig 1A). However, inhibition of Gsk3β is much less potent as a MT stabilizer than Dvl [5]. Here we used neurons from differentiated neuroblastoma (NB2a) cells to further examine the mechanism by which Dvl regulates MT stability. The resistance of MTs to the depolymerizing drug Nocodazole [22] assessed MT stability. Expression of Gsk3β in differentiated neurons does not completely block the MT stabilising function of Dvl (Fig. 1B). Quantification shows that expression of Gsk3β does not increase Mt stability (Fig. 1C), In contrast, Gsk3β induces almost a 30% decrease in the ability of Dvl to stabilise MTs (Fig. 1C). This result suggests that Dvl controls MT dynamics through inhibition of Gsk3β but also through an additional pathway.

### Small Rho GTPases are not required for Dishevelled-mediated microtubule stability

**Figure 2:**
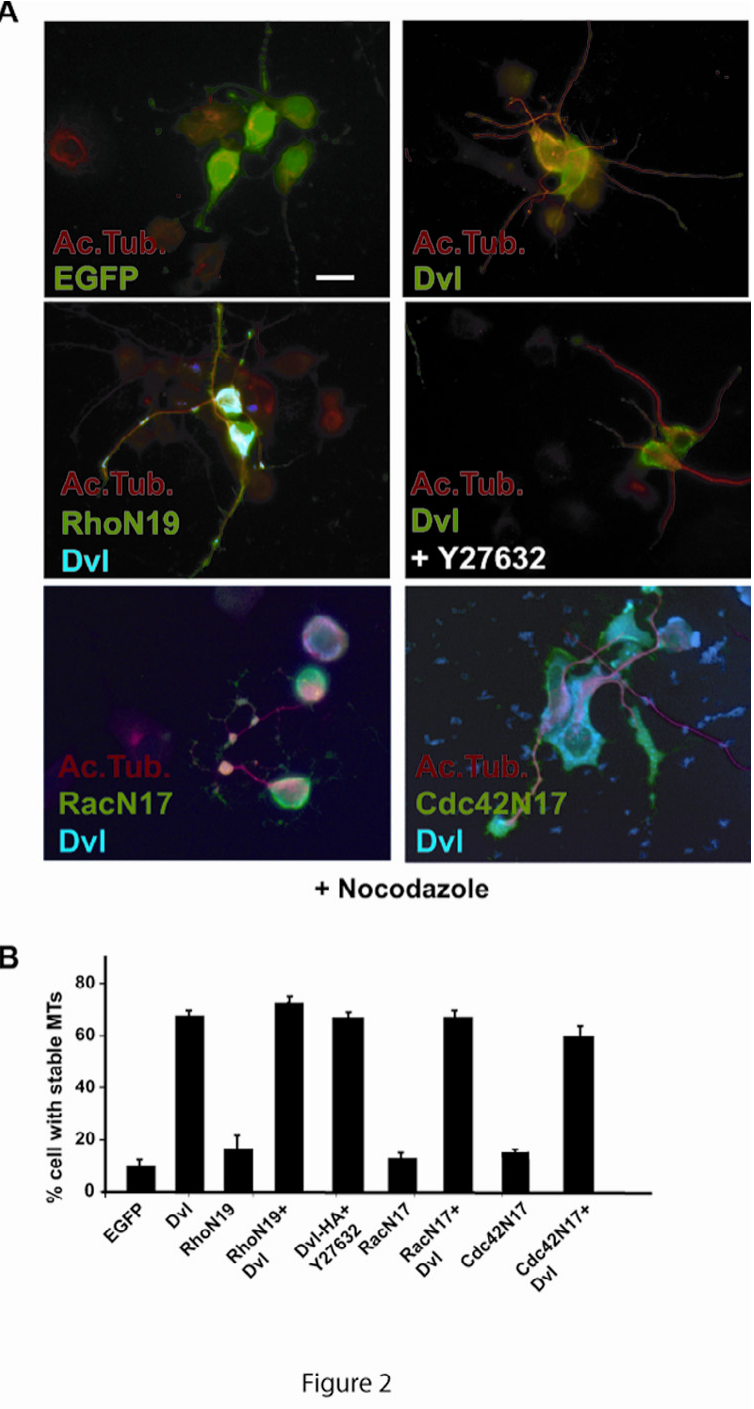
**small GTPases are not required for MT stability induced by Dvl**. **A) **Neurons expressing Dvl alone exhibit increased microtubule stability when compared to control EGFP-expressing neurons. Expression of dominant negative Rho (RhoN19), dominant negative Rac (RacN17), and dominant negative Ccd42 (Cdc42 N17) do not alter the ability of Dvl to stabilise microtubules. In addition, neurons expressing Dvl and treated with the ROCK inhibitor Y27632 exhibit comparable level of microtubule stability as untreated Dvl-expressing neurons. All neurons were treated with Nocodazole to test MT stability **B) **Quantification shows that coexpression of Dvl together with dominant negative Rac Rho, or Cdc42 or the ROCK inhibitor does not change the ability of Dvl to stabilise MTs. Scale bar: 15 μm.

To gain insight into the additional signalling pathways required for the regulation of MT dynamics by Dvl, we turned our attention to the family of small Rho GTPase. WntDvl signalling has been in fact shown to regulate Rho GTPases and these molecular switches have been implicated in MT dynamics [15-21]. RhoA has been previously shown to increase the stability of a population of MTs that are oriented toward the leading edge of migrating cells [16]. Therefore, we examined whether Dvl could regulate MTs through RhoA. We reasoned that if RhoA is a possible downstream effector of Dvl, expression of a dominant negative mutant of RhoA, Rho N19, should block the ability of Dvl to stabilise MTs. As predicted, we found that expression of RhoA in differentiated NB2a neurons increases MT stability (data not shown) whereas expression of RhoN19 alone does not change MT stability compared to control neurons (Fig. 2B). However, neurons expressing both Dvl and RhoN19 still contain MTs after nocodazole treatment (Fig. 2A, B and Additional file 1). Quantification shows that 70% of neurons expressing both Dvl and RhoN19 contain a large number of stable MT similar to neurons expressing Dvl alone (Fig. 2B). To further examine the role of RhoA, we analysed the contribution of ROCK a downstream effector of Rho [23]. Over night treatment with Y27632 a specific inhibitor of ROCK, does not alter the ability of Dvl to stabilise MT (Fig. 2A, B and Additional file 1). In addition, neurons treated with ROCK inhibitor were able to extend processes to a similar extend to control non-treated neurons (data not shown). These results show that Dvl regulates MT stability through a Rho-independent pathway.

We next examined the contribution of Rac on Dvl-mediated MT stability. Although the role for Rac in actin dynamics is very well established, this small GTPase can also regulate the MT network [21,24,25]. In addition, Wnt-Dvl pathway increases the length and branching of dendrites through the activation of Rac1 [26]. We therefore examined whether dominant negative Rac (RacN17) affects Dvl function on MTs. Expression of RacN17 alone dramatically changes the morphology of neurons as cells exhibit shorter and spread processes (data not shown). However, RacN17 does not increase MT stability in NB2a neurons (Fig. 2B). Importantly, neurons expressing both Dvl and RacN17 are still able to retain MTs after nocodazole treatment to similar levels to those neurons expressing Dvl alone (Fig. 2A, B and Additional file 1). These results suggest that Rac1 is not required for the regulation of MT dynamics by Dvl.

Recently Cdc42 has been shown to regulate the MT network by increasing the capture of the plus-ends of MTs through +TIPs proteins such as CLIP170 and the actin binding protein IQGAP [27]. In addition, Cdc42 regulates cell migration through a signalling pathway that required Gsk3β and APC [28]. As both Gsk3β and APC are components of the Wnt canonical pathway, we examined whether Cdc42 is required for Dvl-mediated MT stabilization. Expression of a dominant negative form of Cdc42 (Cdc42N17) does not affect the level of MT stability when compared to control neurons expressing EGFP (Fig. 2B). As observed with Rho and Rac, expression of Cdc42N17 does not affect Dvl function (Fig. 2A and Additional file 1). Quantification reveals that 65% of neurons expressing only Dvl have stable MTs after nocodazole compared to 60% of neurons expressing Cdc42N17 and Dvl (Fig. 2B). These results suggest that Cdc42 is not required by Dvl to regulate MT dynamics. Taken together these findings strongly suggest that Dvl regulates MT stability independently of Rho GTPases.

### Activation of the JNK pathway is required for microtubule stability mediated by Dishevelled

**Figure 3:**
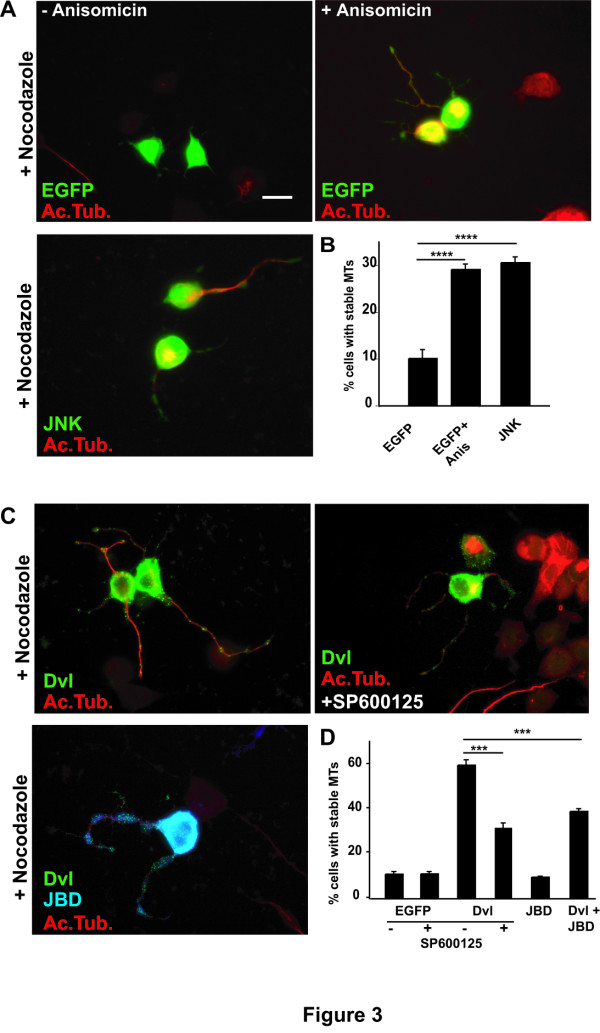
**Activation of JNK induces MT stability while inhibition of JNK blocks MT stability by Dvl**. **A) **EGFP-expressing control neurons treated with low concentration of Anisomicin show higher level of microtubule stability when compared to EGFP-expressing neurons. Neurons expressing JNK alone reveals a level of stability comparable to that one obtained after exposure of neurons to Anisomicin. **B) **Quantification shows that Anisomicin treatment and JNK expression increase the level of MT stability in neurons **C) **Dvl-expressing neurons treated with the JNK inhibitor SP600125 or expressing the dominant negative JNK (JBD) show decreased MT stability when compared to control Dvl-expressing neurons. **D) **Quantification shows that treatment of Dvl expressing neurons with JNK inhibitor SP 600125 or expression of JBD reduces the level of MT stability. Scale bar 15 μm. Three asterisks, *P *< 0.001; For asterisks, *P *< 0.0001.

JNK is an important component of the non-canonical PCP Wnt signalling pathway that regulates cell polarity, cell migration and dendritic development [13,26]. Although JNK has mainly been studied for its role in apoptosis and transcriptional regulation, recent studies have demonstrated that JNK can directly modulate the cytoskeleton. JNK phosphorylates MT-associated proteins such as MAP2 and MAP1B resulting in changes in MT dynamics [29]. In the Wnt signalling pathway JNK is often considered a downstream target of Rac [30,31] but JNK can also act independently of Rac [32]. On the basis of these findings, we investigated the contribution of JNK to Dvl-mediated MT stability. We first examined whether activation of JNK alone was able to induce MT stability. Differentiated NB2a neurons expressing EGFP were treated with Anisomycin, a direct activator of JNK when used a low concentrations [26,33,34]. In the presence of Anisomycin, a significant number of cells contain stable MTs after Nocodazole treatment when compared to controls (Fig. 3A and Additional file 2). Moreover, expression of full-length JNK increases MT stability to comparable levels as Anisomicin-treated neurons (Fig 3A and Additional file 1). Quantification shows that almost 30% of neurons treated with Anisomicin and 32% of neurons expressing JNK have stable MTs in the cell body and along the axon whereas only 8% of control cells contain MTs in the presence of Nocodazole (Fig 3B). This result demonstrates that activation of JNK increases MT stability.

To further test the role of JNK, we asked whether inhibition of JNK affects the function of Dvl in MT stability. SP600125, a specific JNK inhibitor [35] and expression of the JNK dominant negative JBD [36] were used. Inhibition of JNK with SP600125 decreases the ability of Dvl to protect the MT network from Nocodazole to 38% (Fig. 3C, D and Additional file 2). Similarly, expression of JBD decreases the number of Dvl-expressing cells containing stable MT to 40% compare to the 60–65% induced by expression of Dvl alone (Fig. 3C, D and Additional file 2). These results suggest that the inhibition of the JNK pathway blocks Dvl function on MT stability.

**Figure 4:**
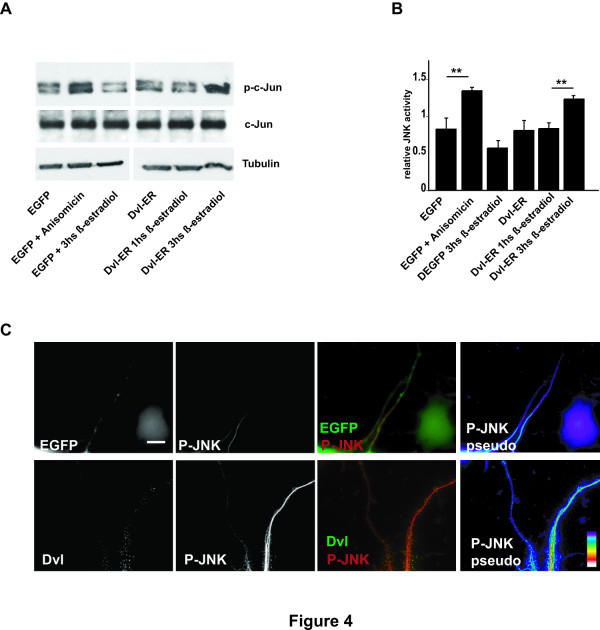
**Dvl expression activates JNK**. **A) **Induction of Dvl-ER expression for three hours with β-estradiol increases the level of p-c-Jun, a read-out of endogenous JNK activity, when compared to controls. Low levels of Anisomicin also increase the level of p-c-Jun when compared with control untreated EGFP-expressing neurons or neurons exposed to β-estradiol for three hrs. However, the levels of total c-Jun remain unchanged. **B) **Quantification of three independent experiments shows that induction of Dvl-ER expression induces a 40% increase in p-c-Jun levels compare to control. **C) **Expression of Dvl increases the level of p-JNK decorating the MT network along neurites when compared to EGFP-expressing control neurons. Pseudocolor panels show Dvl increases the level of p-JNK associated with microtubules. Scale bar 5 μm. Two asterisks, *P *< 0.01.

We then examined whether Dvl can activate endogenous JNK in neurons. The level of phosphorylation of c-Jun was used as a read out of JNK activity [26]. As the activation of JNK might be time dependent, we used the Dvl-ER inducible system in which the levels of active Dvl can be regulated in a time-specific manner. Dvl-ER is a fusion construct in which full length Dvl has been fused to the estrogen receptor such that active Dvl protein is obtained upon induction with β-estradiol [5]. We have previously used this system and found that Dvl becomes active and stabilises MTs in axons after 3 hrs of induction with β-estradiol [5]. Therefore, we examined the levels of phosphorylated c-Jun after different periods of induction. To exclude possible non-specific effects of β-estradiol on MT stability, untransfected control neurons were exposed to β-estradiol for three hrs and the level of p-c-Jun was assessed by western blot (Fig. 4A and 4B). Neurons exposed to β-estradiol exhibit the same level of p-c-Jun as untreated control neurons (Fig 4A and 4B). Treatment of Dvl-ER expressing neurons with β-estradiol for 1 hr does not increase the level of p-c-Jun when compared with EGFP-expressing neurons (Fig 4A and 4B), In contrast, the level of cJun phosphorylation increases by 40% after 3 hrs of β-estradiol treatment (Fig 4A and 4B) to levels comparable to the activation of JNK using Anisomicin (Fig 4A and 4B). Importantly, the level of total c-Jun does not change in both neurons treated with β-estradiol for 3 hrs or Anisomicin when compared to control (Fig 4A). Taken together these results suggest that Dvl activates JNK.

To further examine the role of Wnt-Dvl signalling on JNK activation, we examined the level of active JNK using specific antibodies that detect p-JNK. We decided to test directly the levels of active JNK on MTs along the axons. A specific fixation protocol was used to preserve only the cytoskeleton but removing any cytosolic components. Immunofluorescence analyses show that Dvl colocalises with active JNK on the cytoskeleton (Fig 4C). In addition, Dvl expression increases the level of p-JNK bound to the cytoskeleton when compared to EGFP transfected control cells (Fig 4C). These results strongly suggest that Dvl increases the level of active JNK associated with MTs.

### The DEP domain of Dishevelled is dispensable for signalling to JNK

**Figure 5:**
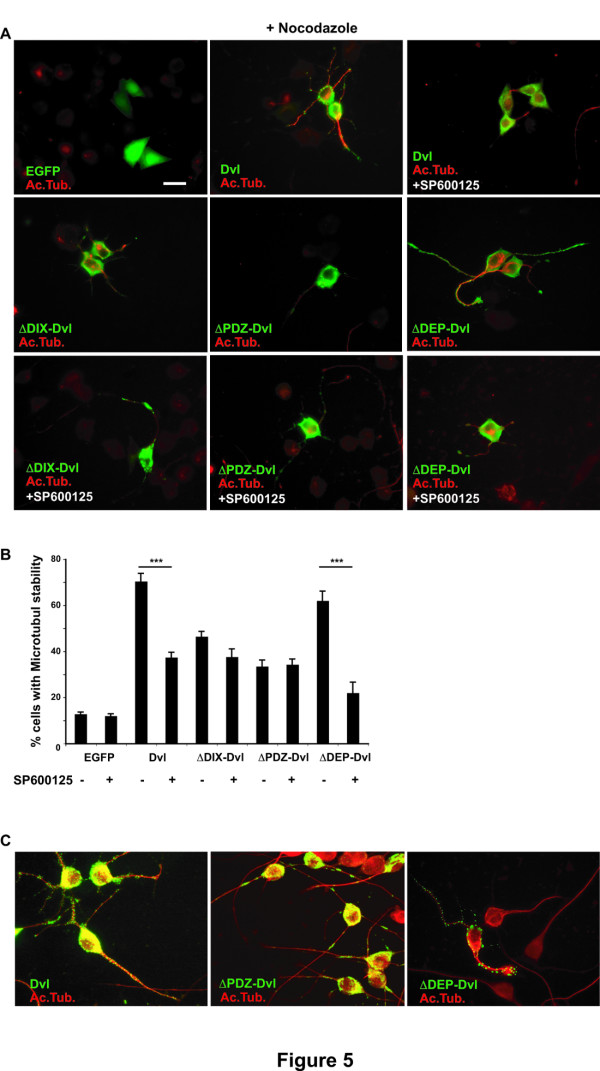
**The PDZ domain and at lesser extent the DIX domains of Dvl are required for activation of JNK**. **A) **Neurons expressing Dvl full-length or Dvl mutants carrying deletions of the DIX, PDZ or DEP domains were treated with or without the JNK inhibitor SP600125 and MT stability was examined after Nocodazole treatment. **B) **Quantification shows that neurons expressing ΔDIX or ΔPDZ mutants and treated with SP600125 exhibit the same low level of MT stability as untreated neurons. Conversely, neurons expressing ΔDEP-Dvl mutant and treated with SP600125 show lower level of MT stability than control untreated neurons. C) Full length Dvl and ΔDEP-Dvl bind to MTs along the axon whereas low levels of ΔPDZ-Dvl mutant remains associated to MTs after detergent fixation. Scale bar 15 μm. Three asterisks, *P *< 0.001.

The Dvl protein has three main conserved domains that are required for signalling through different branches of the Wnt pathway [37,38]. Many studies have shown that DIX and PDZ domains of Dvl are required for the canonical Wnt pathway whereas the DEP domain is required for the PCP pathway [31,39]. However, the correlation between domains and the activation of specific Wnt pathways has proved to be more complex. For example, in some systems the DEP domain is required for the canonical pathway [37,39]. Similarly, the PDZ domain can activate the non-canonical pathway [26,31,38]. In the divergent canonical pathway, the PDZ domain is required for Dvl-Gsk3β mediated MT stability [4]. To begin to understand how Dvl signals through JNK, we have examined the contribution of the different domains of Dvl on JNK-mediated MT stability. NB2a cells expressing full length Dvl and mutant forms of Dvl lacking the DIX, PDZ or DEP domains were examined for their ability to stabilize MTs in the absence or presence of the JNK inhibitor SP600125. We found that neurons expressing ΔDEP-Dvl exhibit similar levels of MT stability to cells expressing full length Dvl (Fig. 5A and 5B). However, inhibition of JNK blocks this effect to control levels (Fig 5A, B and Additional file 3). In contrast, expression of ΔDIX-Dvl and ΔPDZ-Dvl only confers a level of stability of 48% and 38% respectively (Fig. 5A and 5B). Importantly the presence of the JNK inhibitor does not change the level of stability conferred by these two deletion mutants (Fig. 5A, B and Additional file 3). Taken together these results suggest that the PDZ domain and, to a lesser extend the DIX domain, are required for signalling through JNK, whereas the DEP domain is dispensable.

Is the domain responsible for MTs stability also required for the binding of Dvl to MTs? To answer this question we examined the ability of full length and mutant forms of Dvl to bind to MTs. We found that full-length Dvl as well ΔDEP-Dvl and ΔDIX-Dvl remain tightly associated with MTs upon detergent fixation, a protocol that retains MTs associated proteins (Fig. 5C and data not shown). In contrast, ΔPDZ-Dvl mutant remains poorly associated with MTs when compare with ΔDEP-Dvl and Dvl full length (Fig 5C). Taken together these results suggest that the PDZ domain of Dvl is required for MT binding and also for MT stability.

### Dishevelled regulates microtubule stability through two distinct pathways

**Figure 6:**
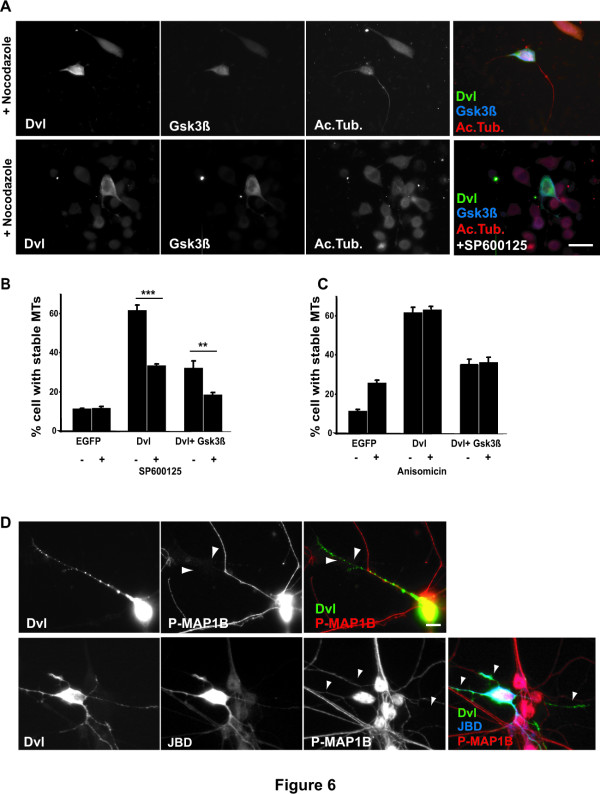
**Dvl signals through two parallel pathways to regulate microtubule stability by Dvl**. **A) **Neurons expressing Dvl an Gsk3β and treated with JNK inhibitor lose the residual level of MTs when compare with neurons expressing Dvl and Gsk3β. Scale bar 15 μm. **B) **Quantification shows that neurons expressing Dvl and Gsk3β and treated with JNK inhibitor have a decreased level of MTs stability than untreated neurons expressing Dvl and Gsk3β. **C) **Quantification shows that neurons expressing Dvl together with Gsk3β and treated with Anisomicin have comparable level of MT stability as untreated neurons. **D) **Inhibition of JNK by expression of JBD does not affect the level of phosphorylated MAP1B in granule cell neurons expressing Dvl when compared to control neurons expressing Dvl alone (arrow). Scale bar 10 μm. Two asterisks, *P *< 0.01; Three asterisks, *P *< 0.001.

We have previously shown that expression of Gsk3β decreases the ability of Dvl to stabilise MTs to 35–40% (Fig. 1C) [4]. We therefore asked whether this residual level of stability is due to signalling through the JNK. We found that inhibition of JNK with SP600125 decreases further the ability of Dvl to stabilise MTs when Gsk3β is already expressed (Fig. 6A and 6B). These results suggest that activation of JNK and inhibition of Gsk3β contribute together to Dvl-mediated MT stability.

Gsk3β and JNK could be part of the same pathway or may act in distinct pathways to modulate MT dynamics. To distinguish between these two alternatives, we tested whether activation of JNK can override the effect of Gsk3β on Dvl function. We found that activation of JNK with Anisomicin does not change the level of MTs stability observed when Dvl and Gsk3β are expressed together (Fig. 6C) suggesting that JNK is not downstream of Gsk3β to regulate MT dynamics.

To examine if JNK is upstream of Gsk3β, we investigated whether JNK affects the levels of Gsk3β-mediated phosphorylation of MAP1B, which is correlated with MT stability and more importantly with the level of Gsk3β activity [11]. For these experiments we used granule cell neurons in which specific antibodies can recognise one of the Gsk3β phosphorylation site in MAP1B [11]. We found that expression of JBD does not affect the ability of Dvl to inhibit MAP1B phosphorylation (Fig. 6D). This result suggests that JNK does not affect Gsk3β activity and therefore it is not upstream of Gsk3β. We then asked if JNK activity was affected by Gsk3β by examining the level of phosphorylation of c-Jun in cells expressing Gsk3β. Western blot analyses show that Gsk3β does not change the levels of phosphorylated c-Jun at either 3 or 16 hrs after transfection (data not shown). Taken together these results suggest that increased MT stability by Dvl is achieved through inhibition of Gsk3β and activation of JNK and that the two pathways are acting independently to regulate the MT cytoskeleton.

## Discussion

Here we show that Wnt-Dvl signalling regulates MT dynamics through two distinct pathways. Dvl induces the concomitant inhibition of Gsk3β and the activation of JNK to increase MT stability. The typical PCP pathway appears not to be involved as small Rho GTPases are not required for this function. These findings highlight a new role for JNK kinase in the modulation of the MT dynamics mediated by Wnt signaling.

We have previously showed that a divergent canonical Wnt pathway regulates MT dynamics. The scaffold protein Dvl is tightly associated with MTs [4] and increases MT stability through the local inhibition of a pool of Gsk3β [5]. In turn, inhibition of Gsk3β by Wnt or expression of Dvl leads to changes in the phosphorylation of MAPs, notably MAP1B, resulting in increased MT stability. Although pharmacological inhibition of Gsk3β increases MT stability, this effect is weaker than that observed by expression of Dvl. Moreover, expression of Gsk3β only partially blocks the ability of Dvl to stabilize MTs [5]. These findings suggest that in addition to Gsk3β, Wnt-Dvl signals through another pathway to regulate MT dynamics.

The family of the small Rho GTPases are not required for the regulation of MT stability upon Wnt activation. Although small Rho GTPases have been extensively shown to control different aspects of actin dynamic and organisation [18,40-43], these molecules can also regulate the MT network. For example RhoA and its effector mDia are necessary for MT stability induced by LPA and activation of integrins [16,44,45]. Conversely Rac and Cdc42 signalling through their effectors PAK and IQGAP respectively are required for the capture of MT plus-end at the leading edge of migrating cells [27] and for inhibition of the MT destabilising effect of Stathmin (OP18) [17,21,46]. Recently Cdc42 and RhoA have been shown to regulate the phosphorylation of Gsk3β to control MT stability and cell polarisation [28,47]. Importantly, the PCP pathway activates small Rho GTPases to regulate tissue polarity, cell migration and dendritic morphogenesis, processes that require the reorganization of the cytoskeleton [26,31,48]. However, expression of Rho, Rac or Cdc42 dominant-negative mutants does not alter the ability of Dvl to stabilise MTs. Thus, Wnt-Dvl signalling regulates MT stability through a Rho GTPase independent pathway.

Wnt signalling regulates MT stability through JNK. In neurons, activation of JNK increases MT stability whereas pharmacological inhibition of JNK or expression of a dominant-negative form of JNK blocks the effect of Dvl on MT stability. In the PCP pathway, JNK is downstream of Rho GTPases [30,31]. However, the finding that small GTPases are not required for MT stabilization by Dvl suggests that activation of JNK is achieved independently of these small GTPases. Interestingly, studies have shown that Dvl can activate JNK in a Rac and Cdc42 independent manner [32]. This is the first demonstration that Wnt signalling regulates MT stability through JNK.

The same domains of Dvl are required for signalling to JNK and Gsk3β to modulate MT dynamics. We have previously shown that the PDZ and DIX domains of Dvl are important for signalling through Gsk3β [4]. Here we show that the PDZ is required for binding of Dvl to the MT network. Moreover, the PDZ and DIX domains are also required for Dvl to signal to JNK. It has been generally accepted that PDZ domain of Dvl is required for the activation of the Gsk3β/β-catenin pathway whereas the DEP domain is required for the PCP pathway [37,39]. However, several studies have shown that the PDZ domain of Dvl is also required for the PCP pathway [26,31,38] suggesting that different domains of Dvl can activate different Wnt signalling pathways depending on the cellular or developmental context. Thus, our finding that Dvl signals to JNK through its PDZ and DIX domains raises the possibility that Gsk3β and JNK act together to regulate MT dynamics.

How does JNK modulate MT dynamics? JNK could regulate MT stability through changes in gene expression or through direct signalling to the cytoskeleton. JNK has been well established as a key regulator of transcription [49,50]. However, we found that Wnt regulates MT stability through a transcription-independent pathway [5] suggesting that the Wnt pathway through JNK directly signals to the cytoskeleton. Consistently, we found that Dvl increases the level of active JNK associated with MTs suggesting that JNK can directly modulate the MT network [51,52] by changing the phosphorylation of MT associated proteins such as MAP2 and MAP1B [29]. Moreover, Wnt signalling regulates the phosphorylation of MAPs suggesting that JNK could mediate these changes. Future studies will elucidate the targets of JNK in this novel Wnt pathway.

Two different pathways that include JNK and Gsk3β contribute to Wnt-mediated effects on MT dynamics. Epistatic analyses show that the concomitant expression of Gsk3β and pharmacological inhibition of JNK strongly blocks the ability of Dvl to stabilize MTs. Interestingly, activation of Gsk3β or inhibition of JNK alone partially blocks Dvl function. These findings indicate that these two kinases are both required to regulate MTs stability via Dvl. Interestingly JNK is not downstream of Gsk3β as activation of JNK does not alter the ability of Gsk3β to block MT stability induced by Dvl. Moreover when JNK is blocked, Dvl is still able to inhibit Gsk3β activity as determined by the level of MAP1B phosphorylation. Conversely when Gsk3β is expressed in neurons the activity of JNK is not altered. Taken together these findings suggest that JNK regulates the cytoskeleton through a distinct pathway that is independent of Gsk3β. In summary, our studies demonstrate a novel role for JNK in Wnt-mediated MT stability and demonstrate that Wnts can simultaneously active different signalling branches to modulate complex cellular processes.

## Conclusion

Here we report that Wnt-Dvl signaling activates two different pathways to modulate MT dynamics. Concomitant inhibition of Gsk3β and activation of JNK mediates the effect of Wnts on MT stability. Interestingly the small Rho GTPases are not required for Wnt-Dvl signaling to the MT network suggesting that an atypical non-canonical pathway is involved in MT stability.

## Methods

### Transient transfections and plasmid constructs

Full length Dvl1, Dvl1 deletion mutants, and Gsk3β were under the control of the CMV promoter [4]. Rho, Rac, Cdc-42 constructs were provided by Dr C. Nobes. Full-length JNK and dominant negative JNK-1 (JBD) construct were kindly provided by Roger Davis. Plasmids for transfection were isolated using a maxi-prep endotoxin-free kit (QIAGEN).

### Cell Cultures and transfection

NB2a cells were maintained in DMEM containing 10% FCS at 37°C and 5% CO2. For immuno-fluorescence analyses cells were cultured on glass coverslips and transiently transfected using Lipofectamin (Invitrogen) for 3 hrs. NB2a cells were then differentiated into neurons by overnight treatment with 1 μM dibutyryl-cyclic-AMP and then fixed For MT stability assay, NB2a neurons were treated with 5 μM Nocodazole for 30 minutes at 37°C and then fixed. To inhibit JNK, neurons were treated with the specific JNK inhibitor SP600125 (Tocris Co) at 10 μM. JNK inhibitor was added 16 hrs after transfection and left for 9 hrs. This period of treatment was chosen because cells transfected with EGFP and treated with SP600125 contain a similar amount of stable MTs to control EGFP expressing cells after Nocodazole treatment. To activate JNK, neurons were treated with Anisomicin (10 mg/ml) 4 hrs before fixation. To inhibit ROCK, neurons were treated with ROCK inhibitor Y27632 (Calbiochem) (100 mM) 3 hrs after transfection and left 15 hrs before fixation.

### Immunofluorescence microscopy

Neurons were fixed in 4% PFA/4% sucrose at room temperature for 20 minutes, permeabilised with 0.02% Triton-X-100 for 5 minutes and blocked in 5% BSA in PBS for 1 hour. To examine the association of proteins to the cytoskeleton, neurons were fixed for 10' at 37C with detergent fixation buffer containing 3% Formaldehyde, 0.2% Gluteraldehyde, 0.2% Triton X 100 and 10 mM EGTA Ph 7.2. Neurons were then permeabilized with 0.02% triton for 2' before blocking. Primary antibodies against acetylated tubulin (Sigma), tyrosinated tubulin (Oxford Biotechnology), HA (Boeringher Mannheim), Myc, Flag (Sigma), phosphorylated MAP-1B (SMI 31) (Affinity BioReagents, Inc) were used. Secondary antibodies Alexa488, Alexa546 and Alexa 350 from Molecular Probes were used.

### Western blots

For western blot analyses, neurons were plated on plastic dishes and 24 hrs after transfection was lysed in RIPA buffer containing 100 μg/ml PMSF, 10 μg/ml aprotinin, 10 μg/ml pepstatin, and 10 μg/ml leupeptin. After protein quantification samples were analysed by SDS-PAGE and western blots. When the inducible Dvl-ER system [53] was used, differentiated neurons were treated with 1 μM β-estradiol for different periods of times. Monoclonal antibody against p-c-Jun (KM-1), c-Jun and tubulin (ab6161) were from Santa Cruz Biotechnology (Santa Cruz CA) and ABCAM respectively. Anti-mouse or anti-rat Ighorseradish peroxidase and chemoluminescence (ECL) solution (Amersham) were used for protein detection. Western blots were scanned, and the optical density of each band was quantified using NIH Image software. Each experiment was done at least 3 times.

### Analyses of microtubule stability

Neurons were transfected with the appropriate plasmids and, 16 hrs after transfection, they were treated with 5 μM Nocodazole for 30 minutes at 37°C, a condition that results in the depolymerization of most MTs in neurons. Stable MTs were stained using an antibody against acetylated tubulin. Neurons containing MTs in both the cell body and along the processes were considered positive for MT stability and given a value of 1 for quantification. Neurons containing low level of MTs or MTs only in the cell body or axon were given a value of 0.5. The percentage of MT stability was defined as the ratio between total number of neurons and those containing MTs. At least 100 cells were counted per experiment and each experiment was repeated at least 3 times.

### Primary neuron isolation and transfections

Cerebella granule cells from neonatal mice were obtained as described previously [54]. Briefly, after dissection, 5 × 10^4 ^cells were plated on coverslips previously treated with 500 μg/ml poly-D-lysine for 1 hour and 30 μg/ml laminin for 3 hrs at room temperature. After 2 days in vitro, neurons were transiently transfected using Lipofectamine 2000 (Invitrogen) for 2 hrs and were cultured for an additional 24 hrs previous fixation.

### Image acquisition and Statistical analysis

Images were captured with an Olympus BX60 or an inverted Zeiss M 200 microscope using a CCD camera (Orca ER), acquired with Metamorph software and processed with Adobe Photoshop. For each experiment more than 100 cells were analysed and the data correspond to at least three different experiments. Data was subject to analysis of variance (ANOVA). Different levels of significances were labelled as followed (For asterisks p < 0.0001; Three asterisks p < 0.001; Two asterisks p < 0.01 and one asterisks p < 0.05).

## Authors' contributions

LC carried out all the experiments, participated in the design of the study and in the draft of the manuscript; PS conceived the study, participated in its design and helped to draft the manuscript. LC and PS have both read and approved the final manuscript.

## Supplementary Material

Additional file 1 single black and white panels of colour merged images of Figure 2 Click here for file

Additional file 2 single black and white panels of colour merged images of Figure 3 Click here for file

Additional file 3 single black and white panels of colour merged images of Figure 5 Click here for file

## References

[B1] da Silva JS, Dotti CG (2002). Breaking the neuronal sphere: regulation of the actin cytoskeleton in neuritogenesis. Nat Rev Neurosci.

[B2] Dent EW, Gertler FB (2003). Cytoskeletal dynamics and transport in growth cone motility and axon guidance. Neuron.

[B3] Galjart N (2005). CLIPs and CLASPs and cellular dynamics. Nat Rev Mol Cell Biol.

[B4] Krylova O, Messenger MJ, Salinas PC (2000). Dishevelled-1 regulates microtubule stability: a new function mediated by glycogen synthase kinase-3beta. J Cell Biol.

[B5] Ciani L, Krylova O, Smalley MJ, Dale TC, Salinas PC (2004). A divergent canonical WNT-signaling pathway regulates microtubule dynamics: dishevelled signals locally to stabilize microtubules. J Cell Biol.

[B6] Schmitt AM, Shi J, Wolf AM, Lu CC, King LA, Zou Y (2006). Wnt-Ryk signalling mediates medial-lateral retinotectal topographic mapping. Nature.

[B7] Liu  Y, Shi  J, Lu CC, Wang ZB, Lyuksyutova AI, Song XJ, Zou Y (2005). Ryk-mediated Wnt repulsion regulates posterior-directed growth of corticospinal tract. Nat Neurosci.

[B8] Lyuksyutova AI, Lu CC, Milanesio N, King LA, Guo N, Wang Y, Nathans J, Tessier-Lavigne M, Zou Y (2003). Anterior-posterior guidance of commissural axons by Wnt-frizzled signaling. Science.

[B9] Hall AC, Lucas FR, Salinas PC (2000). Axonal remodeling and synaptic differentiation in the cerebellum is regulated by WNT-7a signaling [see comments]. Cell.

[B10] Krylova O, Herreros J, Cleverley K, Ehler E, Henriquez J, Hughes S, Salinas P (2002). WNT-3, Expressed by Motoneurons, Regulates Terminal Arborization of Neurotrophin-3-Responsive Spinal Sensory Neurons. Neuron.

[B11] Lucas FR, Goold RG, Gordon-Weeks PR, Salinas PC (1998). Inhibition of GSK-3beta leading to the loss of phosphorylated MAP-1B is an early event in axonal remodelling induced by WNT-7a or lithium. J Cell Sci.

[B12] Logan CY, Nusse R (2004). The Wnt signaling pathway in development and disease. Annu Rev Cell Dev Biol.

[B13] Wallingford JB, Habas R (2005). The developmental biology of Dishevelled: an enigmatic protein governing cell fate and cell polarity. Development.

[B14] Goold RG, Owen R, Gordon-Weeks PR (1999). Glycogen synthase kinase 3beta phosphorylation of microtubule-associated protein 1B regulates the stability of microtubules in growth cones. J Cell Sci.

[B15] Narumiya S, Yasuda S (2006). Rho GTPases in animal cell mitosis. Curr Opin Cell Biol.

[B16] Cook TA, Nagasaki T, Gundersen GG (1998). Rho guanosine triphosphatase mediates the selective stabilization of microtubules induced by lysophosphatidic acid. J Cell Biol.

[B17] Wittmann T, Bokoch GM, Waterman-Storer CM (2003). Regulation of leading edge microtubule and actin dynamics downstream of Rac1. J Cell Biol.

[B18] Nobes CD, Hall A (1999). Rho GTPases control polarity, protrusion, and adhesion during cell movement. J Cell Biol.

[B19] Linder S, Hufner K, Wintergerst U, Aepfelbacher M (2000). Microtubule-dependent formation of podosomal adhesion structures in primary human macrophages. J Cell Sci.

[B20] Tian L, Nelson DL, Stewart DM (2000). Cdc42-interacting protein 4 mediates binding of the Wiskott-Aldrich syndrome protein to microtubules. J Biol Chem.

[B21] Daub H, Gevaert K, Vandekerckhove J, Sobel A, Hall A (2001). Rac/Cdc42 and p65PAK regulate the microtubule-destabilizing protein stathmin through phosphorylation at serine 16. J Biol Chem.

[B22] Hoebeke J, Van Nijen G, De Brabander M (1976). Interaction of oncodazole (R 17934), a new antitumoral drug, with rat brain tubulin. Biochem Biophys Res Commun.

[B23] Fujisawa K, Fujita A, Ishizaki T, Saito Y, Narumiya S (1996). Identification of the Rho-binding domain of p160ROCK, a Rho-associated coiled-coil containing protein kinase. J Biol Chem.

[B24] Watabe-Uchida M, John KA, Janas JA, Newey SE, Van Aelst L (2006). The Rac activator DOCK7 regulates neuronal polarity through local phosphorylation of stathmin/Op18. Neuron.

[B25] Eaton S, Wepf R, Simons K (1996). Roles for Rac1 and Cdc42 in planar polarization and hair outgrowth in the wing of Drosophila. J Cell Biol.

[B26] Rosso SB, Sussman D, Wynshaw-Boris A, Salinas PC (2005). Wnt signaling through Dishevelled, Rac and JNK regulates dendritic development. Nat Neurosci.

[B27] Fukata M, Watanabe T, Noritake J, Nakagawa M, Yamaga M, Kuroda S, Matsuura Y, Iwamatsu A, Perez F, Kaibuchi K (2002). Rac1 and Cdc42 capture microtubules through IQGAP1 and CLIP-170. Cell.

[B28] Etienne-Manneville S, Hall A (2003). Cdc42 regulates GSK-3beta and adenomatous polyposis coli to control cell polarity. Nature.

[B29] Chang L, Jones Y, Ellisman MH, Goldstein LS, Karin M (2003). JNK1 is required for maintenance of neuronal microtubules and controls phosphorylation of microtubule-associated proteins. Dev Cell.

[B30] Moriguchi T, Kawachi K, Kamakura S, Masuyama N, Yamanaka H, Matsumoto K, Kikuchi A, Nishida E (1999). Distinct domains of mouse dishevelled are responsible for the c-Jun N-terminal kinase/stress-activated protein kinase activation and the axis formation in vertebrates. J Biol Chem.

[B31] Habas R, Dawid IB, He X (2003). Coactivation of Rac and Rho by Wnt/Frizzled signaling is required for vertebrate gastrulation. Genes Dev.

[B32] Li L, Yuan H, Xie W, Mao J, Caruso AM, McMahon A, Sussman DJ, Wu D (1999). Dishevelled proteins lead to two signaling pathways. Regulation of LEF-1 and c-Jun N-terminal kinase in mammalian cells. J Biol Chem.

[B33] Iordanov MS, Pribnow D, Magun JL, Dinh TH, Pearson JA, Chen SL, Magun BE (1997). Ribotoxic stress response: activation of the stress-activated protein kinase JNK1 by inhibitors of the peptidyl transferase reaction and by sequence-specific RNA damage to the alpha-sarcin/ricin loop in the 28S rRNA. Mol Cell Biol.

[B34] Coffey ET, Hongisto V, Dickens M, Davis RJ, Courtney MJ (2000). Dual roles for c-Jun N-terminal kinase in developmental and stress responses in cerebellar granule neurons. J Neurosci.

[B35] Bennett BL, Sasaki DT, Murray BW, O'Leary EC, Sakata ST, Xu W, Leisten JC, Motiwala A, Pierce S, Satoh Y, Bhagwat SS, Manning AM, Anderson DW (2001). SP600125, an anthrapyrazolone inhibitor of Jun N-terminal kinase. Proc Natl Acad Sci U S A.

[B36] Dickens M, Rogers JS, Cavanagh J, Raitano A, Xia Z, Halpern JR, Greenberg ME, Sawyers CL, Davis RJ (1997). A cytoplasmic inhibitor of the JNK signal transduction pathway. Science.

[B37] Yanagawa S, van Leeuwen F, Wodarz A, Klingensmith J, Nusse R (1995). The dishevelled protein is modified by wingless signaling in Drosophila. Genes Dev.

[B38] Penton A, Wodarz A, Nusse R (2002). A mutational analysis of dishevelled in Drosophila defines novel domains in the dishevelled protein as well as novel suppressing alleles of axin. Genetics.

[B39] Axelrod JD, Miller JR, Shulman JM, Moon RT, Perrimon N (1998). Differential recruitment of Dishevelled provides signaling specificity in the planar cell polarity and Wingless signaling pathways. Genes Dev.

[B40] Pollard TD, Blanchoin L, Mullins RD (2000). Molecular mechanisms controlling actin filament dynamics in nonmuscle cells. Annu Rev Biophys Biomol Struct.

[B41] Ridley AJ, Hall A (1992). The small GTP-binding protein rho regulates the assembly of focal adhesions and actin stress fibers in response to growth factors. Cell.

[B42] Ridley AJ, Paterson HF, Johnston CL, Diekmann D, Hall A (1992). The small GTP-binding protein rac regulates growth factor-induced membrane ruffling. Cell.

[B43] Kozma R, Ahmed S, Best A, Lim L (1995). The Ras-related protein Cdc42Hs and bradykinin promote formation of peripheral actin microspikes and filopodia in Swiss 3T3 fibroblasts. Mol Cell Biol.

[B44] Palazzo AF, Cook TA, Alberts AS, Gundersen GG (2001). mDia mediates Rho-regulated formation and orientation of stable microtubules. Nat Cell Biol.

[B45] Palazzo AF, Eng CH, Schlaepfer DD, Marcantonio EE, Gundersen GG (2004). Localized stabilization of microtubules by integrin- and FAK-facilitated Rho signaling. Science.

[B46] Wittmann T, Bokoch GM, Waterman-Storer CM (2004). Regulation of microtubule destabilizing activity of Op18/stathmin downstream of Rac1. J Biol Chem.

[B47] Eng CH, Huckaba TM, Gundersen GG (2006). The Formin mDia Regulates GSK3{beta} through Novel PKCs to Promote Microtubule Stabilization but Not MTOC Reorientation in Migrating Fibroblasts. Mol Biol Cell.

[B48] Tao W, Pennica D, Xu L, Kalejta RF, Levine AJ (2001). Wrch-1, a novel member of the Rho gene family that is regulated by Wnt-1. Genes Dev.

[B49] Karin M (1995). The regulation of AP-1 activity by mitogen-activated protein kinases. J Biol Chem.

[B50] Gupta S, Barrett T, Whitmarsh AJ, Cavanagh J, Sluss HK, Derijard B, Davis RJ (1996). Selective interaction of JNK protein kinase isoforms with transcription factors. Embo J.

[B51] Yujiri T, Fanger GR, Garrington TP, Schlesinger TK, Gibson S, Johnson GL (1999). MEK kinase 1 (MEKK1) transduces c-Jun NH2-terminal kinase activation in response to changes in the microtubule cytoskeleton. J Biol Chem.

[B52] Yujiri T, Ware M, Widmann C, Oyer R, Russell D, Chan E, Zaitsu Y, Clarke P, Tyler K, Oka Y, Fanger GR, Henson P, Johnson GL (2000). MEK kinase 1 gene disruption alters cell migration and c-Jun NH2-terminal kinase regulation but does not cause a measurable defect in NF-kappa B activation. Proc Natl Acad Sci U S A.

[B53] Smalley MJ, Sara E, Paterson H, Naylor S, Cook D, Jayatilake H, Fryer LG, Hutchinson L, Fry MJ, Dale TC (1999). Interaction of axin and Dvl-2 proteins regulates Dvl-2-stimulated TCF-dependent transcription. Embo J.

[B54] Lucas FR, Salinas PC (1997). WNT-7a induces axonal remodeling and increases synapsin I levels in cerebellar neurons. Dev Biol.

