# Supplementary figures and images for: c-Jun N-terminal kinase (JNK) cooperates with Gsk3β to regulate Dishevelled-mediated microtubule stability
Source: BMC Cell Biol. 2007 Jul 3;8:27. doi: 10.1186/1471-2121-8-27 (PMC1940000; doi:10.1186/1471-2121-8-27)

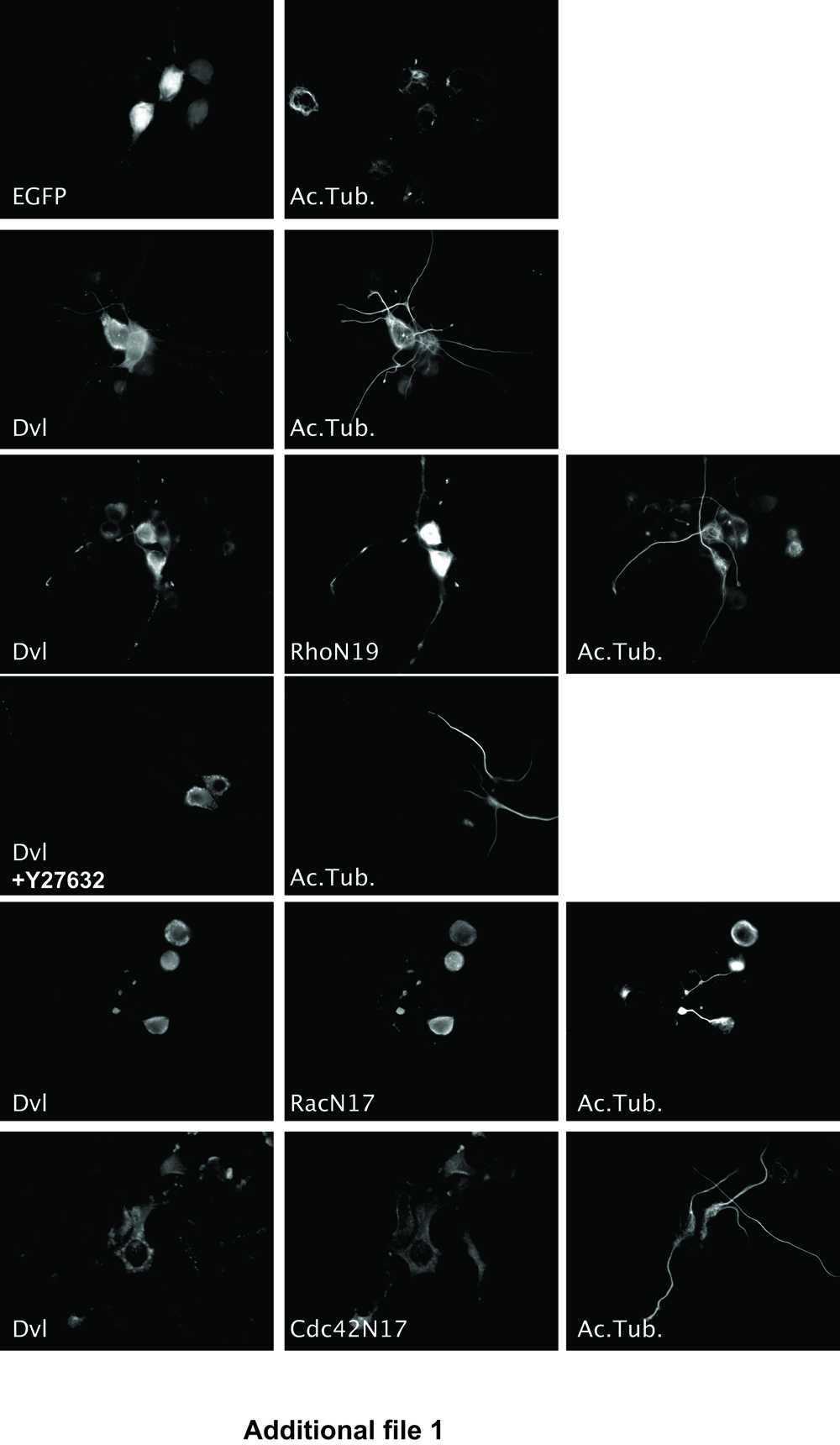

Supplement: Additional file 1 — single black and white panels of colour merged images of Figure 2 [file 1471-2121-8-27-S1.tiff]

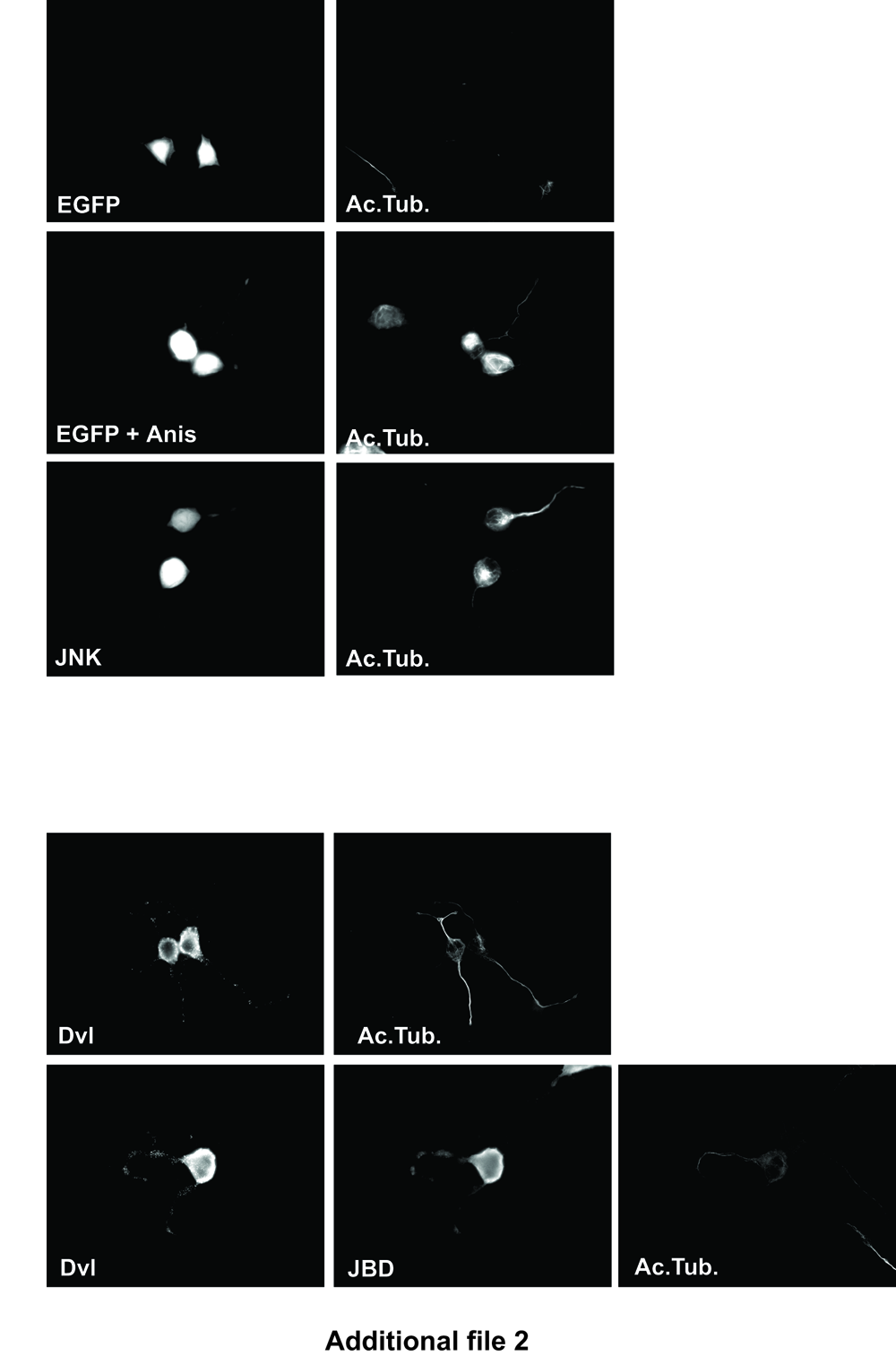

Supplement: Additional file 2 — single black and white panels of colour merged images of Figure 3 [file 1471-2121-8-27-S2.tiff]

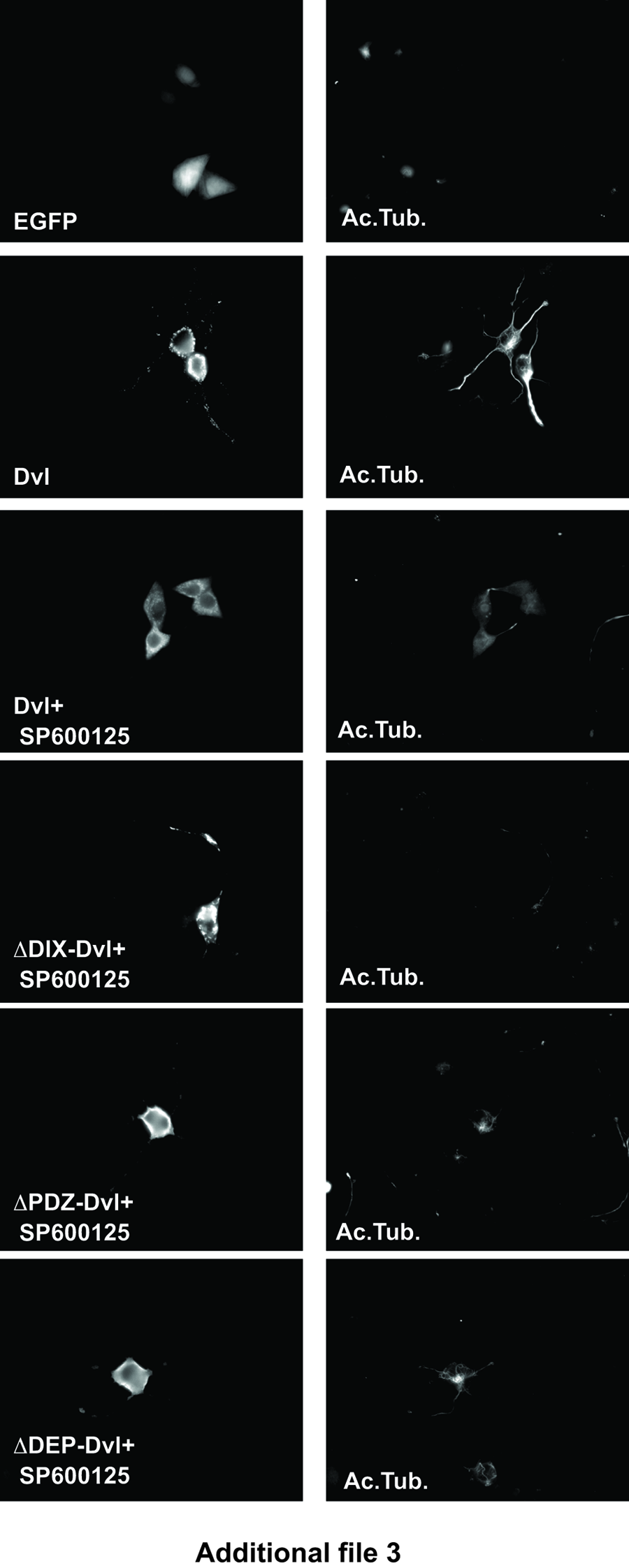

Supplement: Additional file 3 — single black and white panels of colour merged images of Figure 5 [file 1471-2121-8-27-S3.tiff]
